# Social norms and family planning decisions in South Sudan

**DOI:** 10.1186/s12889-016-3839-6

**Published:** 2016-11-22

**Authors:** Sumit Kane, Maryse Kok, Matilda Rial, Anthony Matere, Marjolein Dieleman, Jacqueline EW Broerse

**Affiliations:** 1 KIT Health, Royal Tropical Institute, Mauritskade 63, Amsterdam, 1090 HA The Netherlands; 2 Independent Consultant, Wau, Western Bahr el Ghazal South Sudan; 3 School of Public and Environmental Health, University of Bahr el Ghazal, Wau, Western Bahr el Ghazal South Sudan; 4 Athena Institute for Research on Innovation and Communication in Health and Life Sciences, Faculty of Earth and Life Sciences, VU University Amsterdam, Amsterdam, The Netherlands

## Abstract

**Background:**

With a maternal mortality ratio of 789 per 100,000 live births, and a contraceptive prevalence rate of 4.7%, South Sudan has one of the worst reproductive health situations in the world. Understanding the social norms around sexuality and reproduction, across different ethnic groups, is key to developing and implementing locally appropriate public health responses.

**Methods:**

A qualitative study was conducted in the state of Western Bahr el Ghazal (WBeG) in South Sudan to explore the social norms shaping decisions about family planning among the Fertit community. Data were collected through five focus group discussions and 44 semi-structured interviews conducted with purposefully selected community members and health personnel.

**Results:**

Among the Fertit community, the social norm which expects people to have as many children as possible remains well established. It is, however, under competitive pressure from the existing norm which makes spacing of pregnancies socially desirable. Young Fertit women are increasingly, either covertly or overtly, making family planning decisions themselves; with resistance from some menfolk, but also support from others. The social norm of having as many children as possible is also under competitive pressure from the emerging norm that equates taking good care of one’s children with providing them with a good education. The return of peace and stability in South Sudan, and people’s aspirations for freedom and a better life, is creating opportunities for men and women to challenge and subvert existing social norms, including but not limited to those affecting reproductive health, for the better.

**Conclusions:**

The sexual and reproductive health programmes in WBeG should work with and leverage existing and emerging social norms on spacing in their health promotion activities. Campaigns should focus on promoting a family ideal in which children become the object of parental investment, rather than labour to till the land — instead of focusing directly or solely on reducing family size. The conditions are right in WBeG and in South Sudan for public health programmes to intervene to trigger social change on matters related to sexual and reproductive health; this window of opportunity should be leveraged to achieve sustainable change.

## Background

After a long civil war (1955–2005), South Sudan became an independent country in July 2011. The war has destroyed much of the public infrastructure, and economic activities and opportunities are few. The newfound freedom and peace have been regularly disrupted by violent civil conflict in some parts of the country. The health care system is also weak, with severe shortages of health workers and functioning health facilities [1, 2]. As a result, South Sudan has one of the world’s worst population health indicators; this is particularly so for sexual and reproductive health (SRH). For instance, at 789 deaths per 100,000 live births, it has one of the highest maternal mortality ratios (MMR) in the world [3]; similarly, the contraceptive prevalence rate (CPR) is just 4.7%, with only 1.7% of women reporting using modern methods [4, 5]. While reliable data disaggregated by state and ethnic group are not available, it is reasonable to expect that, minor differences notwithstanding, the situation is similar in all 28 states. South Sudan’s SRH challenges relate both to the supply and demand sides of SRH services.

In this context, and with the purpose of informing the development of a locally appropriate intervention approach, a study was conducted to explore factors influencing SRH-related behaviours and decision-making on a range of SRH issues, including ‘family planning’, in Western Bahr el Ghazal (WBeG) state. Evidence [6] shows that increased contraceptive use alone has “cut the number of maternal deaths in developing countries by about 40% over the past 20 years” [7]. Since 2011, unlike some other parts of South Sudan, WBeG has been relatively peaceful, and at the time of the study, some forms of basic health and reproductive health services, including modern contraceptives, were generally available across the state. Thus, demand-side, population-level factors are perhaps as important as the supply-side factors for the low CPR in WBeG.

South Sudan is home to more than 50 ethnic groups; at the national level, the Dinka and the Nuer constitute the biggest ethnic groups. While they constitute a sizeable part of the population in South Sudan, in some states other ethnic groups tend to predominate. For instance, in WBeG, the three main ethnic groups are the Fertit, Luo (or Jur) and Dinka; the Fertit, a moniker used to refer to a loose conglomeration of more than 23 non-Dinka, non-Arab, non-Fur and non-Luo people, are the predominant group [8]. Unlike the Dinka and the Nuer, who are pastoralists, the Fertit are predominantly agriculturist people involved in subsistence farming. The Fertit, like all other South Sudanese ethnic groups, are patriarchal; men have the power to decide on all aspects of the family and in society at large, and women’s position is subordinate to men [9–11]. Edwards [12] argues that a range of societal, historical and political processes have led to a situation where gender inequalities in South Sudanese society have become entrenched and disadvantage women in social, economic and political realms alike.

No matter where one is, planning a family is a complex process, with the couple’s decisions regarding family size, timing of pregnancy/spacing and contraceptive use affected by a variety of factors. According to de Francisco et al.’s [13] conceptual framework, a range of interlinking factors in the household, community, larger society and the political environment shape the SRH related decisions and actions of individuals; these factors also shape the consequences experienced by individuals of their decisions and actions. According to the framework, the intimate, family and social relations, including intra/inter-generational relations and gender relations, shape individuals’ ability to make SRH related decisions. These close interpersonal relationships are set within an intermediate circle of kinship structures and community institutions, which are, in turn, nested in an outer circle of national political and social institutions, power structures and ideologies. Within these overlapping spheres of influence, individuals and social groups occupy positions of relative advantage or disadvantage with respect to their access to information and other resources — including their capacity to make decisions; this has important implications for their own and others’ SRH and rights. A wide range of influences shape both behaviour and opportunities, with consequences for SRH-related behaviours (decisions and actions); they point out that these influences are transmitted through community-level institutions. For instance, the meaning and value given to what constitutes sexual health, reproductive health, satisfaction, distress, motherhood and fatherhood is always strongly influenced by dominant cultural norms. Similarly, social norms also create powerful ideals of manhood, womanhood, masculinity and femininity, and they define what sexual and reproductive behaviour is appropriate for men and for women, at different stages of life. Social norms condemn or condone SRH-related behaviours, expectations and decision-making processes; they also define access to resources and information, which together are necessary for decision-making related to health (including SRH).

While factors influencing SRH-related behaviours and decisions include both those related to availability and access to services and social- and individual-level factors, the focus of this paper is on the latter. The paper provides insight into how social norms shape behaviours and decisions related to family planning among the Fertit people in WBeG. Such insight can be useful for public health policymakers and programmers in WBeG for designing and implementing locally appropriate and culturally sensitive SRH interventions; this insight can also be valuable for other states in South Sudan with similar, large agriculturist communities.

## Methods

A qualitative exploratory study was conducted. Data were collected through focus group discussions (FGDs) and semi-structured interviews (SSIs) conducted with a variety of purposefully selected informants, as detailed in Table 1. The following sections further explain the sampling and recruitment principles and processes.

**Table 1:** Overview of study participants and data collection

Method	Profiles of study participants	Number of activities (number of participants)
FGD	Community members: Female 18–35 years (Not in union^a^)	1 (8)
	Community members: Female 18–35 years (In union)	1 (8)
	Community members: Male above 35 years	1 (8)
	Community members: Male 18–35 years	1 (8)
	Health workers	1 (6)
SSI	Community member: Female 18–35 years (Not in union)	5
	Community member: Female 18–35 years (In union)	6
	Community member: Male 18–35 years	6
	Community member: Female above 35 years	6
	Community member: Male above 35 years	4
SSI with Key Informants	Traditional birth attendants	4
	Traditional leaders	3
	Health facility personnel	5
	State SRH managers	2
	NGO representatives	3

^a^Participants were either In Union or Not In Union at the time of the study; we articulate relationship status this way because in WBeG, one would publicly state one’s status as married only if the relationship was formalised either in a traditional ceremony, or in the church. However, for the sake of convenience we use the terms married/unmarried in the paper

Topic guides for FGDs and SSIs were developed using de Francisco et al.’s [13] conceptual framework. The topic guides included questions exploring social norms and beliefs about sex, sexuality, roles and relations between men and women, reproduction, and what shapes the decision-making on matters related to reproduction. The topic guides also included questions about preferences and expectations from, and views about, current SRH services. The topic guides for health and other workers included questions along the same lines, but with a view to exploring the situation from their perspective. The FGD and SSI topic guides for community members were prepared in English and translated into Wau Arabic (by investigators MR and AM). The topic guides were defined further during the initial stakeholder workshops, pre-tested in the study site and also adapted iteratively as the study progressed. The FGDs and SSIs with community members were conducted in Wau Arabic, a language spoken by all around Wau, including the Fertit people; interviews with health and other workers were conducted in Wau Arabic or English, depending on the preference of the health worker.

The analytical framework provided by the theory of planned behaviour (TPB) [14, 15] was used to critically analyse factors shaping behaviour and decision-making related to family planning among the Fertit people in WBeG. According to TPB, three major antecedent domains influence a person’s intention to perform a behavior: 1) attitude towards and belief that performing the behaviour will lead to the desired outcomes; 2) social norms related to the behavior; and 3) one’s perceived control over or perceived ability to perform the specified behaviour. The TPB contends that a positive attitude and positive outcome expectations alone are not enough to shape decisions and behaviour; the two domains, the prevalent social norms and one’s beliefs about own ability and capacity to act, also operate concomitantly to affect individuals’ decisions and actions. The TPB is a mid-range theory which has been widely used and is well suited to describe the antecedents of particular behavioural intentions [16]. Recognizing that in many situations individuals and groups defy what appear to be strong social norms [17], and that norms both shape actions of agents and are at the same time themselves being constantly shaped by these actions, we draw on the critical realist explanatory tradition to go one step further to discuss and explain norm congruence, norm defiance and, thereby, norm maintenance or transformation [18]. To do so, we draw on Archer (1998, Ch 14, p20) [17], who argues for an analysis which approaches structure and agency through “analytical dualism”, wherein “the structural, cultural and agential components are analyzed separately, with a focus on their logical relations and the conditions and possibilities that these allow”. The analytical emphasis is thus twofold: explaining how the social structures shape the actions and interactions of individuals, and how at the same time the social interactions between agents also shape the social structures and social relations, both maintaining or reproducing and transforming them.

### Study sites

The study was conducted in Wau county in the state of WBeG in South Sudan. Two locations were selected based on the homogeneity of the residents (all Fertit). Further, the locations were also within the coverage area of health services, particularly SRH services. This was important, as the geographical coverage of health services remains poor in many parts of WBeG. Finally, the two locations represented two different settings in Wau county: one in Wau town and the other a rural area. The *a priori* assumption behind choosing these two locations was that perhaps within the same social group the way norms related to behaviour and decisions might be moderated differently in different settings.

### Sampling, recruitment of study participants and data collection

The main categories of study participants are summarized in Table 1. Community members were purposefully selected with the help of village elders, health workers from a local non-governmental organization (NGO) and the county health department. Among community members, only those aged 18 years and above were included in this study; a separate but linked study has been conducted among adolescents. We purposefully categorized participants into those between 18 and 35 years and those above 35 years — the assumption being that the former would be more subject to the norms related to sexuality and reproduction, and the latter would be the ones involved in enforcing the norms, shaping preferences, setting expectations and influencing the decision-making and health-seeking behaviours of the former.

Data collection began with FGDs among community members to identify different aspects of the subject, and differences in views among participants on the subject. This was followed by SSIs to obtain more in-depth understanding. For FGDs with community members, participants were homogenous in terms of age and marital status, yet diversity was sought in terms of social and economic status (based on: inputs from elders related to social identity, ownership of assets such as bicycles, level of education). FGD participants were not involved in the SSIs.

Health facility personnel working in the local health centre of the study sites were included in the study. First, an FGD was conducted to identify different aspects of the subject, and differences in views among health workers on the subject. Participants included a clinical officer, two nurses, a health assistant and two community health workers. The FGD was followed by SSIs with those personnel specifically responsible for reproductive health at the health centre. FGD participants were not involved in the SSIs.

Key informants were also purposefully selected for inclusion in the study; they were selected based on their active SRH-related role within the health system and the study community, and identified through the initial stakeholder consultations. Key informants included traditional leaders, traditional birth attendants, state- and county-level SRH service managers and NGO representatives. Given the serious shortage of health and social workers in South Sudan, the pool of managers and NGO representatives was small — in fact there was only one SRH-related officer at both county and state health department level, and both were interviewed. Similarly, all three NGO representatives working on SRH in Wau county were interviewed.

Data were collected between October 2014 and April 2015, from three visits to Wau. FGDs and SSIs with community members, traditional leaders and traditional birth attendants were conducted by research team members who hailed from the study area, were fluent in the local language and had experience in conducting qualitative research; interviewers and participants were matched by sex. FGDs and SSIs with health workers, managers and NGO representatives were done in English. Data were collected until data saturation was reached and no new insight emerged; this was possible to assess, as at the end of each day of data collection, the research team debriefed and discussed the emerging findings. In total, 5 FGDs (with 38 participants) and 44 SSIs were conducted. This is congruent with the general experience on saturation; according to Creswell [19], a sample size of around 30–50 is generally sufficient to achieve analytical saturation in a qualitative study.

### Data analysis

SSIs and FGDs were digitally recorded, translated from Wau Arabic into English (where applicable) and transcribed verbatim; the translations were independently checked. Analysis of the transcripts was carried out using a comprehensive thematic matrix to facilitate the identification of common patterns and trends arising from the narratives, using NVivo 10 software. This was done in parallel by three researchers (SK, MK, MR), and emergent conceptual categories were arrived at through a process of argumentation and consensus. Validity of findings and of the analysis was further assured through a data validation workshop (*n* = 10) and interviews with key informants (*n* = 2), and also through follow-up interviews with some (*n* = 4) of the study participants in both study sites. The daily debriefing sessions and insights from these validation interviews and workshop were also used to develop and further clarify emerging analytical themes.

### Ethical considerations

The study was approved by the Independent Ethics Committees of KIT Royal Tropical Institute, Amsterdam, and the national Ministry of Health of the Government of South Sudan. Administrative approval was given by the WBeG Ministry of Health. Informed consent was taken from all participants. Consent was sought only after the person had been contacted to participate (and had in principle agreed), but before any of the interview questions were asked. For those who could read, the consent form was given to them and also read out to them to seek both their written and oral consent. For those who could not read, the consent form was read out to them, and their consent was recorded. Confidentiality was maintained throughout, and steps were taken to anonymize the data and to minimize risk of accidental disclosure and access by unauthorized third parties.

Sex, sexuality and reproduction are sensitive, intimate and yet social issues. At the beginning of the consent process, participants were informed of their right to refuse to answer any questions they might find intrusive. The interviewers were also very conscious of this, and did not press ahead with a line of inquiry if they noticed the participant was not comfortable. Furthermore, given the sensitive nature of the topic, there is a risk of opening up hitherto closed, yet painful chapters and experiences in the person’s life. To ensure support if such a situation arose, a trained counsellor was available, as were medical referral services. No such situation requiring counselling or medical referral emerged during data collection. However, there were many instances of people in the community seeking help to get treatment for individuals, and this was provided — for example, on two occasions, the research team used its car to take a child and his mother to the state hospital for further treatment.

## Results

Findings are presented along three broad lines: knowledge of and attitudes to pregnancy, family planning and contraceptive use; social norms shaping family planning decisions; and participants’ perceived control over or perceived ability to make reproductive decisions and choices.

### Knowledge of and attitudes to pregnancy, family planning and contraceptives

#### Childbearing as God’s will and one’s duty

Both Fertit women and men consider having children very important. Getting pregnant after marriage was looked on positively, and a common belief across sexes and across age groups is that pregnancy is ‘God’s will’:

*“Pregnancy is of course from God … pregnancy is from God.” (B FGD M under 35)*

*“Once you get married … if God wills you give birth right away as that is the main reason.” (FGD Males over 35)*

*“Only one of my three daughters has no child. Only one … God did not give her a child.” (FGD Females over 35)*

Another related view shared by men and women alike, albeit perhaps more so by the older generation, was that it was desirable to have as many children as possible — that it was a woman’s duty to bear children:

*“Our community believes that bearing a child is from God. If God gives you strength to give birth to 10 or 12 you will be lucky.” (Traditional Leader)*

#### Spacing pregnancies, the right thing to do?

All respondents recognized that a sufficient amount of time was necessary between two pregnancies, and had a favourable view of spacing. They believed this for many reasons; a common belief among women was that getting pregnant immediately after delivery was bad for the unborn child:

*“… When a woman gives birth and before her baby sits she is pregnant again this is when you harm your small baby…” (Female over 35)*

Another belief was that having frequent pregnancies was bad for the woman’s health:

*“And when it is frequent it will affect the uterus and the pelvis will be tired.” (Female Married under 35)*

*“Housework and food also will be difficult. If a woman gives birth every year it wears her out.” (FGD Females Married under 35)*

Men, both young and old, also knew about the importance of spacing; their arguments, however, tended to be more related to the negative effect of frequent pregnancies on the recently born child or on the yet unborn child. In an FGD some older men pointed out, perhaps referring to a local belief, that getting the wife pregnant again before the first child has started walking was detrimental to the health of the unborn child. As the following quotes illustrate, this understanding and a favourable attitude to spacing was shared by the younger men:

*“From the time your wife delivers a baby and when it starts walking, then you can go on top of his/her mother again so that you can bring another child. But if the child born is not yet walking, if you get on top of his/her mother, that child will be paralyzed.” (FGD Males over 35)*

*“Sometimes if there is a baby in the stomach and there is [already] a [breastfeeding] child, the [unborn] child can get dehydrated in relation to the child’s breastfeeding … the [unborn] child will be … sometimes you will find a child like this hand of mine … will be very skinny…” (FGD Males under 35)*

Men and women pointed out how this understanding about the importance of spacing was part of their traditional knowledge about pregnancy; a traditional leader echoed this thus:

*“For our old generation we used to wait even after two years…* O*ur traditions are against that [back-to-back pregnancies].” (Traditional Leader)*

Some older women argued that the times were harder now, that raising children was more difficult now, and that this made it necessary for women to space pregnancies:

*“Child delivery in the past is not like the present. If you bear ten children, where will they get education…? This is not good. It is better to bear children and have gaps between them so that they can get good education. But if you have ten, nine, five children, there will be many, and raising them becomes very difficult.” (FGD Females over 35)*

Others disagreed, pointing out that because childbearing was God’s will, one could not say how many children one should have:

*“Childbirth is from God. I cannot say they should have this many children.… I don’t like it; I think it is very bad. How can they stop a woman from having a child? God has given that to you. Are they going to stop it?” (FGD Female over 35)*

Younger women also had some misgivings; for instance, during an FGD among younger women, there was much agreement when one of the participants said that if a woman does not have a child for three or four years, it can make subsequent deliveries more difficult. A discussion among younger women during an FGD went as follows:

*“If you stay long without getting pregnant it becomes hard, so three years is reasonable… Four years is too long, and some places will be tight, and it puts women in a dangerous situation … So three years is reasonable.” (FGD Females Married under 35).*

#### The use of contraceptives

As the following quotes illustrate, younger women, unlike other informants, had a uniformly favourable attitude towards using contraceptives, perhaps reflecting a more pragmatic understanding of the situation. They not only know about modern contraceptive options but also commonly use them, although some women mentioned they had stopped using contraceptives after experiencing negative physical effects:

*Facilitator: “Do you know how to plan your family?”*

*Respondent: “Family planning is like taking pills every morning. These pills are given. If you go to the health centre and you say that you want to plan pregnancy, they give you pills … if the injection does not suit you. There is an injection given for up to six months. So you need to plan pregnancy.” (Female Married under 35)*

*“Contraceptive pills are very good if you can tolerate it. I tried contraceptive pills before and I used to see my menses twice a month. This is why I stopped it.” (Female Unmarried under 35)*

While all recognized the importance of spacing, and were knowledgeable about modern contraceptive options, there were disagreements about their use. Men were mixed in their views about women using contraceptives to space pregnancies. While some took a more pragmatic view, others, including some younger men, strongly disapproved:

*“For younger girls who had children early and do not want to become pregnant again, the doctor can offer contraceptive pills … The woman can be given injections or contraceptive pills to prevent pregnancy, and young girls can go back to school.” (Male Married over 35)*

*“Those condoms, those pills, those injections. Our fathers in the past didn’t do it. For what reason should we come to do it? Does it mean that we alone do not know how to plan a family? My wife is not going to swallow pills or get injection … for what?” (FGD Males Married over 35)*

Many men were concerned about women going behind their backs and using contraceptives, particularly the long-acting injectables; while not explicit, there was insinuation that the health services were somehow abetting this. They argued that it was because of this trend that some men were forbidding their wives to use modern contraceptives:

*“Some women do it in agreement with their husbands; others just go to pharmacies on their own and buy the pills and use it without telling their husbands … the men do not know what the woman is doing; they have no ideas about such things. Such men think it is God who has not blessed them with another baby.” (Male Married under 35)*

Health facility personnel shared concerns regarding how some men perceived the promotion of contraception — as attempts by outsiders to deny them their right to have many children, as outsiders were the ones promoting contraceptive use. The following quote highlights the importance of handling delicately any intervention to promote contraceptive use.

*“… Let the youth give birth because a long time ago we gave birth too, and they would say you are denying their children to have children… They say before we used to give birth, why now does the white man want our girls not to give birth?” (FGD Health Personnel)*

In one of the FGDs, young women recognized that many men had a suspicious attitude towards the health services. They agreed that this was not the right thing to do and that such a situation would make things difficult for everyone:

*“Family planning is good, but … planning should be done after you agree with your husband. You tell him that life is difficult … other women just go and start family planning without involving their husband, and then it causes problems in the house; the woman stops the planning, and then she starts giving birth one after the other. So this is a mistake.” (FGD Females Married under 35)*

### Social norms on childbearing, spacing and contraceptive use

The theory of planned behaviour refers to social norms as structural powers which shape people’s intentions and behaviour. Cialdini et al. [20] and others [21] argue that when studying the influence of norms on human behaviour, it helps to try to distinguish between descriptive and injunctive norms, even if sometimes it is empirically difficult. Descriptive norms refer to individuals’ beliefs about the prevalence of a particular behaviour and about what most (relevant) others do in a particular situation. Injunctive norms, on the other hand, refer to the extent to which individuals perceive that influential (and relevant) others expect them to behave in a certain way, and to perceive that social sanctions will be incurred if they do not. This section presents findings on how social norms, both injunctive and descriptive, shape the Fertit people’s intentions and behaviours about spacing and contraceptive use.

#### Social norms on marriage and childbearing

Among the Fertit (and most ethic groups in South Sudan), the injunctive social norm around marriage is that a man marries a woman to be able to bear children, to replace dead family members. There is social pressure on women to bear children, and not bearing children incurs social disapproval, even ostracism.

*“The community is the main reason for all this, especially neighbours, friends and parents. They complain a lot that their son needs to have babies to replace a dead uncle or a dead grandfather, so they want him to name relatives who passed away.” (Health Personnel)*

*“Yes, they insist on what they are doing. Regarding family planning, some say I married this woman … why shouldn’t she give birth. This woman must give birth and not take any contraceptives.” (NGO)*

As the following interaction during an interview with a young man illustrates, it is normative that women have children, and that asking a wife to stop is just not done:

*Facilitator: “Are you married?” Respondent: “Yes.” Facilitator: “Did you ever think or did you ever ask your wife not to become pregnant?” Respondent: “No, I have never done that.”*

*Facilitator: “Why?” Respondent: “I have never thought about that before.” (Male Married under 35)*

*“Our community does not encourage spacing children.” (Male under 35)*

In fact the social norm is that if a woman does not bear children, then she, as the quote below illustrates, is considered not worth keeping. Further, men are also normatively expected to have multiple children. Those who do not continue to father children run the risk of being labelled as infertile and subjected to ridicule; they also risk their wives leaving them for other men.

*“If the wife just stays for six months without getting pregnant, immediately they start asking ‘Was she brought just to go to the toilet, and who is to pay for that?’.” (Health Personnel)*

*Facilitator: “What does the community say about a family where the woman does not become pregnant for a while?”*

*Respondent: “The community does not speak well about that. They will say that the woman is not fertile and the man is wasting his time. He should go and find another woman who can bear him some children. Sometimes the woman’s family will say that the man is infertile and their daughter needs to find another man. Some of them will start having an affair.” (Male Married under 35)*

Thus, injunctive social norms on marriage and childbearing have a major influence on the intentions and behaviours of men, women and couples about spacing and contraceptive use.

#### Social norms on spacing

In Fertit society, there is an injunctive social norm that women should get rest after each pregnancy. As the following quote from an older woman illustrates, while having many children is desirable and expected, both at the societal and the individual level, women and men reported that in society it is frowned upon if a woman in the family becomes pregnant very soon after childbirth:

*“When a woman gives birth and two months later she gets pregnant again, it is shameful. All your family, even your own mother, will be blamed, because they will say why did you let this girl get pregnant and her baby is still small. It is a bad reputation in the family.” (Female over 35)*

One traditional leader highlighted the social sanctions in the form of shaming of the family and the woman if a baby were to be born with a low birthweight because of insufficient spacing:

*“Our traditions are against that [back-to-back pregnancies]; if a woman gives birth to an immature child [with low birthweight], she will be called ‘Na-Ngoyo’ … means the mother of an immature child. It is a shame in our community.” (Traditional Leader)*

It was also clear that there were no injunctive social norms which explicitly or implicitly sanctioned the use of modern contraceptives:

*Facilitator: “So there are no traditions that prohibit contraceptives?”*

*Respondent: “No. If you go for an injection, it is up to you ….” (Female Unmarried under 35)*

*“You from your own will and the will of your wife. If you see this woman having [multiple and frequent] childbirths, like for me, maybe I will go to the doctor to give us family planning.” (FGD Males under 35)*

Men and women relied on the actions and experiences of important others (descriptive norms) to inform their own intentions and actions; the important others influencing contraceptive decisions and choice included family and close friends, and the traditional leaders. Women’s attitudes to different forms of modern contraceptives were informed by experiences of friends and family members:

*“This issue of contraception … even me, I wanted it. I have my sister-in-law who has an IUD inserted, and it is giving her a hard time: every month she bleeds a lot. So I decided not to take anything and just save myself and pray to God to save me.” (Female over 35)*

### Ability to act and decide

Kabeer [22], in her influential work on women’s empowerment, frames women’s “ability to define one’s goals and act upon them” as their ‘agency’. Agency is exercised in relation to others; as Kabeer [21] explains, it is “more than about just observable action” and includes the ability to negotiate and bargain, subvert, resist and manipulate, and also more intangible cognitive processes of reflection and analysis (1999: p 438). This section shows how Fertit women’s ability to decide about their pregnancies and spacing of pregnancies is constrained. It also shows how they are using the opportunities available to them to subvert and resist and overcome these constraints — a testimony to the dynamic and relational nature of human agency and how it also shapes social norms.

#### Entrenched patriarchy and women’s constrained agency

Women consistently referred to ‘abstinence’ after delivery as the way to avoid getting pregnant soon after; in an FGD among married young women, the respondents explained how they went about getting their husbands to cooperate — a poignant reflection of the severely constrained nature of women’s agency and of their resigned attitude to the social acceptance of their husbands having sex outside their marriage, despite being well aware of the risks of contracting sexually transmitted infections:

*“If you have a three-month-old baby and your husband goes and finds another chance, let him go. Tell him to find someone who will not bring us disease and who will join me and we talk and laugh. In this way, your baby will not suffer.” (FGD Females Unmarried under 35)*

*“You will stay away from the husband a bit. You can allow your husband to go around like when you have a child in your hand.” (FGD Females Married under 35)*

Entrenched patriarchy among the Fertit bestows on men the status of the head and the sole decision-maker of the household; not only do the men and their families uphold and operate within this framework, the entrenched patriarchy operates such that women themselves measure and express their freedom of choice within this acceptable framework. According to many of the male informants, both young and old:

*“The woman cannot decide more than the man.” (Male Unmarried under 35)*

*“This decision comes from the man. (…) it is the man who will decide.” (Male Married over 35)*

*“It is the man. How he does it … he must tell her the reality that life is difficult.” (Male Married under 35)*

Women’s acceptance of these socially bounded and constructed cultural expectations reflects the extent to which their ability to decide about their reproductive lives is constrained in WBeG. Women’s acceptance of this unequal social order, and of the finality with which they accept their constrained agency, is illustrated by the following quote:

“*The decision comes from the man. Our relatives see that birth allows inheritance, and if you do not want to give birth, men do not agree. They should be the ones to take the decision because he is the person in charge. He is like the president of the house or the chief of the house. He is the one to see if his wife should give birth every year or after how many months, whether it will affect her health or affects upbringing of children or the way they live at home.” (Female Married over 35)*

A young woman pointed out, *“If it is the woman who says that she wants to stop, they* [men and society] *take it in a different way.” (Female Married under 35)*

#### Subverting the hegemony, covertly and overtly

This constrained agency, however, is not going unchallenged; both men and women, young and old, are questioning the appropriateness and the continued feasibility, particularly economic feasibility, of the current social order. The return of peace presents opportunities; unlike before, people now see opportunities beyond just subsistence agriculture and survival. Earlier, children were seen as extra hands to till the land, and the responsibility of the parents was to provide food and shelter. Young men and women recognize these changing economic realities; they appreciate the responsibility and importance of investing in children’s education; and also that one should have only as many children as one can afford to provide good education for.

*“Schools are expensive, and it is good to have sex in such a way that she does not get pregnant.” (Male Married under 35)*

Some young men are also calling for the need for a partnership approach to deciding about family and family planning:

*“This family planning depends on two sides. You, the man, and the woman: this is an agreement between you, of course.” (FGD Male under 35)*

As discussed above, women are covertly defying this unequal social order. As the quotes below illustrate, some women are also doing this overtly, taking matters into their own hands, often to the chagrin of men and other women, and demanding a say on issues affecting their lives. A young woman added:

*“You tell him* [the husband]*, but if it is a husband that will cause problems, you don’t tell him. Some men will not accept it. They don’t want their wives taking contraception. … Yes,* [then] *you go along* [secretly] *and inject or take the pills.” (Female Unmarried under 35)*

All three traditional leaders interviewed were strongly of the view that the man should and does decide on all matters related to reproduction in the household. However, as the quote below indicates, the challenge mounted by some women to this domination by covertly and overtly taking charge of their reproductive lives is triggering a rethink among the Fertit elders and shaping a new normality:

*“Couples are supposed to agree together on when to produce children, but in many cases women will say they have been told in health facilities that it is not yet time for a child. This is what is frustrating many men in families.” (Traditional Leader)*

This is also along the lines of what some health facility personnel and reproductive health service managers indicated: that they have recently noticed a change in the way women and couples approach the matter — perhaps, as indicated earlier, a change that is driven by the new economic realities:

*“It is only this year that I see women starting to say they do not want to give birth. But before this was not there; they had the appetite for childbirth. Now because life has become very difficult … this is when I saw women start to come and ask for the injection and pills.” (Health Personnel)*

## Discussion

Consistent with the theory of planned behaviour [13], we found that a positive attitude and positive outcome expectations about spacing of pregnancies alone are not enough to shape decisions and behaviour; the prevalent social norms and one’s beliefs about one’s capacity to act also operate concomitantly to affect decisions and actions. The findings above show, and we discuss further in this section, how social norms shape the agency and actions of individuals, and how at the same time, broader changes in society, the social interactions between agents and their agency also shape the social norms, both maintaining and reproducing or transforming them.

### The multifaceted influence of social norms on procreation decisions

Findings clearly show that while both men and women desire to have many children, they have a good knowledge of the importance and benefits of spacing pregnancies and of using modern contraceptive methods to do so. This knowledge and positive attitude towards spacing is, however, failing to translate into decisions to use contraceptives among the Fertit in WBeG. Two overlapping explanations emerge from our findings. On the one hand, social norms around pregnancy and childbearing and the entrenched patriarchal privileges intersect to concentrate and maintain decision-making powers in the domestic, economic and public realms in men’s hands, and constrain Fertit women’s agency in the reproductive realm. On the other hand, our findings also recognize that men’s agency in the reproductive realm is perhaps similarly constrained by these social norms and by the very hegemonic patriarchy that privileges men. These findings are consistent with the evidence that use of contraceptives and other SRH services is not merely a matter of knowledge and rational choice but is mediated by social norms and power relations based on gender and ethnicity [23, 24]. They are also consistent with the large body of anthropological and sociological literature supporting the view that couples’ reproductive decisions are negotiated within gender-based power relations and within the context of local social norms and health systems [25–28]. In line with our findings, and the hegemonic patriarchal social situation notwithstanding, many caution against a universally tyrannical representation of men’s roles in the reproductive realm, arguing that such a representation is both inaccurate and unhelpful [23, 29]. While the findings above indicate that patriarchy has been reinforced by the violent and fragile environment of South Sudan, they also show how it is being questioned and challenged, both by women and men.

### Competing social norms: an opportunity to help define a new normality

For Fertit men and women, young and old, in urban and rural settings alike, having children and expanding one’s family is an important social expectation, and people desire to have children [30]. Similar to other patrilineal and patrilocal societies in sub-Saharan Africa, marriage is a key social institution, and its primary function is ‘childbearing’, with women seen as a means of reproduction [31, 32]. These social norms around reproduction remain strong and entrenched among the Fertit people of WBeG, South Sudan. However, the nature of social norms is such that conformity is conditional: people would stop conforming to a norm if there were doubts or disagreements about what the norm seeks to enforce or if it cannot be enforced. Evidence shows that no matter how permanent and rigid norms might appear, in any society, competing norms are constantly at odds with each other, and norms are constantly evolving, being negotiated and being replaced by other collective beliefs about which behaviours are appropriate in society in the evolving context [33, 34]. Our findings show that Fertit men and women are challenging patriarchal social expectations, questioning, testing and transgressing the boundaries set by existing social norms and in the process opening windows of opportunity for redefining normality in WBeG society. In addition, in both urban and rural areas, the descriptive social norm of having as many children as possible is under competitive pressure from two other social norms: the injunctive norm on spacing pregnancies, and the injunctive norm that one must take good care of children. Unlike before, when providing food and shelter was what primarily entailed providing care, nowadays, good care is understood to involve providing good education to one’s children. People also increasingly recognize that they can bear the responsibility and the cost of providing good education to only a few, and not many, children.

### The post-conflict period and opportunities for renegotiating the social compact

Lianos [35] has explored the social processes of conflict, post conflict and emergence of peace as aetiliogies of macro- and micro-level social change. Lianos [35] argues that individuals, groups and other actors adjust their strategies to make the most of the new situation, thus participating in enhancing the legitimacy of the emerging new conditions. Conflict, post conflict and peace constitute social change, and social actors develop strategies to navigate it and benefit from it. This change, catalyzed by the disruption of the traditional social order as a result of the civil war, the chronic insecurity, fragility and the internal displacement, paradoxically offers women and men opportunities and resources to subvert entrenched norms and hegemonies. To some extent, the findings of this study indicate that the new political and economic realities of the post-conflict setting, and the return to peace, might be catalysing the norm change processes in South Sudan. The return to peace, and South Sudanese society’s transitions from a militaristic male-dominated society to a society now focused on nation building and with aspirations of progress, with improving access to knowledge and services, is also opening up opportunities for women and creating spaces for the renegotiation and reconfiguration of socio-political relations; and in the process also emboldening men and women to challenge the hegemonic order.

### Navigating change: enabling women to exercise agency

Many Fertit, including men, do not support the status quo. However, some men, in both urban and rural areas, are wary of their women clandestinely using contraceptives. If the impression that health services are encouraging this becomes commonplace, the SRH programme in WBeG state may become entangled in the complex gender and power dynamics within society — to the detriment of women. While recognizing that social institutions such as the health services are gendered spaces, which reflect and reproduce gender inequalities in society, we argue that the WBeG SRH programme must take explicit and immediate action to prevent such an impression from emerging among the menfolk and society at large. SRH services should take care to ensure that women’s agency is not undermined in such a process, and instead work towards creating safe spaces for women to exercise their agency. There seems to be some openness to joint decision-making on reproductive matters; this is a window of opportunity to promote both gender equality and reproductive health. An explicit gender-transformative approach [36–38] that includes interventions which promote dialogue among couples, family members and society at large, and which builds on social norms around the importance of women to be able to get ‘rest between pregnancies’, could be a feasible and effective way forward. Such an approach could also apply to other settings in South Sudan where the situation is similar to WBeG.

### Limitations

The study has some limitations. Based on the conceptual framework, we expected generational hierarchies and some significant others to be important influences on decisions on sex, sexuality and reproduction. We did not find any explicit evidence of this. While men and women did refer to elders as shaping their decisions, we did not find anyone being particularly influential (e.g., mother-in-law, father, aunt). It is possible that indeed within the Fertit society, many people shape and influence decisions on these matters, and not just a few significant others; it is also possible that our data collection somehow fell short and that we have failed to identify these influences sufficiently.

The topics of sex, sexuality, reproduction and decision-making on these matters are sensitive subjects. There is a risk that people hesitate to talk openly or that they only give socially desirable answers. These constraints were anticipated, and steps were taken to loosen them. Preparatory trips were made to familiarize the study team with the WBeG context and the study sites. Much effort was put into identifying research collaborators, one male and one female, who not only spoke the local language and had experience of qualitative research but were also Fertit themselves. Preliminary visits were made to the study sites before the actual data collection, to meet the villagers and the elders, explain the nature of the study and effectively seek the village’s consent; these visits were village gatherings and, essentially, elaborate confidence-building exercises. As regards the risk of socially desirable answers, the researchers who conducted the interviews with community members know the Fertit culture well and were aware of such a risk. Furthermore, during daily debriefing sessions we involved a local resource person who is knowledgeable about Fertit society, its traditions and its social norms generally, and on matters related to SRH, to make better sense of research participants’ accounts; her involvement served as both a quality check and also an additional level of insight. Finally, the overwhelming interest and the frank interaction we encountered during data collection, and given that the Fertit are not shy about talking about sex and sexuality, makes us confident of the validity of the study findings.

## Conclusions

While the social norm which expects people to have as many children as possible remains well established among the Fertit community of the state of WBeG in South Sudan, it is under competitive pressure from other existing norms which make spacing of pregnancies socially desirable, and from emerging norms on what entails taking good care of one’s children. The latter is changing: the focus is moving from looking at children as labour, to investing in them and providing them with a good education. People increasingly recognize that they should only have as many children as they can afford to educate well. The long war has weakened or disrupted the existing social norms in South Sudan. The return of peace and stability and the emergence of a new economic order are creating opportunities for Fertit men and women to challenge and reconfigure social norms on childbearing and family planning. The public health programmes in WBeG should work with and make use of existing and emerging social norms on spacing and caring for children in their health promotion activities. Instead of focusing directly or solely on reducing family size, campaigns should focus on promoting a family ideal in which children become the object of parental investment.

We argue that the conditions are right in WBeG and in South Sudan to trigger social change on matters related to SRH, and that the post-conflict environment of South Sudan and its people’s aspirations for freedom and a better life offer an opportunity to intervene to change social norms, including but not limited to those affecting reproductive health, for the better; this opportunity should be leveraged to achieve sustainable change.

## Abbreviations

CPR: Contraceptive prevalence rate
FGD: Focus group discussion
SRH: Sexual and reproductive health
SSI: Semi-structured interview
TPB: Theory of planned behaviour
WBeG: Western Bahr el Ghazal
